# Combined microbiome and metabolome analysis of Dacha and Ercha fermented grains of Fen-flavor Baijiu

**DOI:** 10.1016/j.fochms.2025.100298

**Published:** 2025-09-11

**Authors:** Dingwu Qu, Yurong Wang, Lubo Cao, Qiangchuan Hou, Zhongjun Liu, Ji'an Zhong, Zhuang Guo

**Affiliations:** a Brewing Technology Industrial College, Hubei University of Arts and Sciences, Xiangyang, Hubei, China; b Hubei Provincial Engineering and Technology Research Center for Food Ingredients, Hubei University of Arts and Science, Xiangyang, Hubei, China; c Xiangyang Lactic Acid Bacteria Biotechnology and Engineering Key Laboratory, Hubei University of Arts and Science, Xiangyang, Hubei, China; d Xiangyang Liquor Brewing Biotechnology and Application Enterprise-University Joint Innovation Center, Xiangyang, Hubei, China; e Xiangyang Fen-flavor Baijiu Biotechnology Key Laboratory, Xiangyang, Hubei, China

**Keywords:** Light-flavor Chinese liquor, Lactic acid bacteria, Multi-omics integration, Ethyl acetate, Flavonoid biosynthesis

## Abstract

Fen-flavor Baijiu is produced via two fermentation rounds (Dacha and Ercha), and quality is shaped by microbes in fermented grains. We hypothesized that the two rounds select distinct lactic acid bacteria (LAB) consortia with different metabolic potentials that associate with stage-specific metabolites and flavor compounds. We profiled 24 fermented-grain samples using shotgun metagenomics and untargeted metabolomics. Ercha showed lower alpha-diversity and a composition distinct from Dacha. *Lactobacillus acetotolerans* dominated Dacha, whereas *Acetilactobacillus jinshanensis* dominated Ercha. We detected 225 differential metabolites; 12 involved in flavonoid biosynthesis were higher in Dacha, while pyrimidine metabolism was more prominent in Ercha. Several LAB species—including L. *acetotolerans*, *Lentilactobacillus hilgardii*, *Lactobacillus amylovorus*, and *Lactobacillus amylolyticus*—showed positive correlations with these flavonoids. Genes encoding L-lactate dehydrogenase and acetate kinase were mainly carried by L. *acetotolerans* and associated with acetic acid and ethyl acetate in fermented grains. These outcomes supported our hypothesis and suggested actionable levers for production: stage-targeted monitoring of marker taxa/genes and rational starter design to steer flavor formation in Fen-flavor Baijiu.

## Highlights

• *Lactobacillus* and *Acetilactobacillus* dominated late-stage Fen-flavor Baijiu.
• *L. acetotolerans* dominated Dacha; *A. jinshanensis* dominated Ercha.
• Twelve flavonoids were higher in Dacha among 225 differential metabolites.
• Flavonoid biosynthesis was enriched in Dacha; pyrimidine metabolism in Ercha.
• LAB taxa carried key genes for starch, ethanol, and ester formation.

## 1 Introduction

Fen-flavor is one of the three primary Chinese Baijiu flavors and is recognized as the most prestigious ancestral Baijiu flavor type (Luo et al., 2023). Traditional Fen-flavor liquor is produced through a unique process that involves “cleaning stubble, fermentation in ground tanks, and double steaming” to yield various base liquors, each with unique characteristics and styles. The first fermentation product, known as “Dacha,” is obtained through the fermentation of sorghum and has a pronounced fragrance, a mellow and sweet entrance, and a subtle grain aroma. The second fermentation product, called “Ercha,” has a slightly milder fragrance, a spicy entrance, and a longer aftertaste (Yang et al., 2024). After these two types of liquors are evaluated, graded, and stored, they are blended to obtain the final Baijiu according to the specific quality and grade requirements. Therefore, the control of flavor quality during these two stages of base wine production is crucial for determining the overall quality of Baijiu.

The flavor compounds of Baijiu hold the key to its aroma, style, and quality. The volatile compounds of Fen-flavor Baijiu are relatively numerous and complex. For example, a total of 79 volatile compounds and 1596 metabolites have been identified in Fen-flavor Baijiu in previous studies (Tian et al., 2024). Recently, researchers compared the aroma characteristics of four different grades of Fen jiu and identified 70 volatile compounds that can serve as aroma markers for predicting the quality of the final wine (Li et al., 2023). In addition to ethyl acetate and ethyl lactate, trace compounds such as β-damarone, geosmin, and other aroma compounds have also been found to play an important role in determining the quality of Fen-flavor Baijiu (Wang et al., 2022). Notably, the brewing process used for Baijiu differs according to the region of production, and the composition of flavor substances can also vary significantly among liquors of the same flavor type (He et al., 2020). However, most previous studies on Baijiu have focused on the flavor compounds present in the finished wine, and research on the metabolite differences between fermented grains has been rather limited.

The production of Chinese Baijiu relies on natural fermentation. During this process, the microorganisms from Daqu, raw materials, and the environment contribute to the production of various flavor compounds (Chai et al., 2021; Fu et al., 2021; Zhou et al., 2024). Microbial metabolites are the main sources of flavor during Baijiu brewing, and the generation of flavor compounds such as esters, alcohols, and acids is species- or even strain-dependent (Pang et al., 2021). Owing to the inherent features of “ground tank fermentation,” the fermentation microorganisms for Fen-flavor Baijiu are largely derived from fermented grains (Tu et al., 2022). Thus, it is important to analyze the structure and function of the microbial communities present in these fermented grains to study the mechanisms of flavor formation in the base liquors of Fen-flavor Baijiu. However, despite the significance of the fermentation microbiota in influencing liquor quality, our understanding of the characteristics and functions of microorganisms in fermented grains during Fen-flavor Baijiu fermentation remains limited.

Recent advancements in metagenomic technology (Ko et al., 2022) have greatly enhanced our ability to analyze bacterial community structures alongside their functions. Coupling these tools with untargeted metabolomics enables linkage of community composition, genetic potential, and flavor-relevant metabolites in Dacha and Ercha fermented grains. We hypothesized that the two fermentation rounds harbor distinct lactic acid bacteria–centered microbiomes with different metabolic potentials that shape stage-specific metabolite profiles and key flavor compounds, and that dominant taxa contribute genes for enzymes involved in acid and ester formation (e.g., L-lactate dehydrogenase and acetate kinase). To test this, we analyzed 24 Dacha and Ercha fermented-grain samples using shotgun metagenomics and untargeted metabolomics to map microbial succession, metabolic potential, and associations with flavor formation.

## 2 Material and methods

### 2.1 Sample collection

**Fig. 1:**
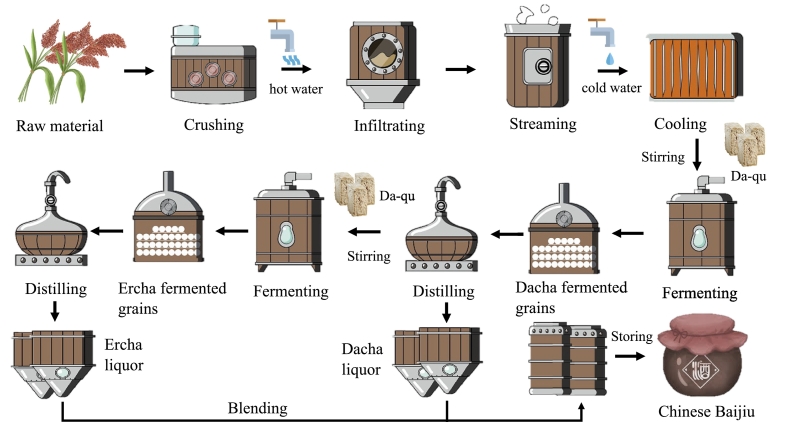
Flow chart showing the traditional process of Fen-flavor Baijiu production.

Twenty-four fermentation tanks from the brewing workshop of a brewing company in Xiangyang City, Hubei Province, were selected by stratified simple random sampling. The sampling frame comprised all ground tanks operating under standard conditions and at the target fermentation time (Dacha: 28 days; Ercha: 21 days) on the sampling date (Fig. 1); tanks under maintenance or with recorded process deviations were excluded. Eligible tanks were stratified by fermentation round, assigned unique IDs from the workshop register, and 12 tanks per stratum were selected without replacement using a computer-generated random sequence (R; fixed seed). All tanks were 1.2 m deep. From each selected tank, a 500 g fermented-grain core was collected at 0.6 m depth at the tank center using a soil sampler. Large-crop (Dacha) samples were labeled DC1–DC12 and second-crop (Ercha) samples EC1–EC12. Samples were placed in a cryogenic box, transported to the laboratory within 3 h, and stored at −80 °C. Each tank constituted one biological replicate (*n* = 12 per group; total *n* = 24).

### 2.2 DNA extraction and metagenomic sequencing

DNA was extracted from fermented grains using the QIAGEN DNeasy mericon Food Kit (Qiagen, Hilden, Germany) based on the manufacturer's instructions. DNA integrity, size, and concentration were evaluated through 1 % agarose gel electrophoresis and NanoDrop spectrophotometry (Thermo Fisher Scientific, Waltham, MA, USA). Sequencing libraries were constructed on the Illumina NovaSeq 6000 platform at Novogene Company (Beijing, China). Paired reads, 150 bp in length, were generated in both the forward and reverse directions. After quality control, high-throughput sequencing was performed as previously described (Cai et al., 2021). No technical replicate libraries were generated; downstream analyses were performed per sample (*n* = 24).

### 2.3 Analysis of metagenomic sequencing data

Raw sequences were filtered using fastp (version 0.23.2) software to remove the adapter and low-quality sequences. On average, each sample provided 5.35 Gb of high-quality genomic DNA free of contamination from plant and human sources. Species-level information was obtained using Braken software (v2.9). HUMAnN3 (version 3.6) was employed to annotate metagenomic functional features and metabolic pathways based on the UniRef90 database. Megahit (version 1.2.9) software was used to assemble shotgun readings into contigs, and the assembly sequences with a length of less than 500 bp were filtered out. Using prodigal (version 2.6.3) (options: -p meta), a prokaryotic gene prediction software, functional genes could be identified and predicted based on these assembly sequences. Predicted gene sequences from multiple samples were combined, and a non-redundant gene set with 95 % identity and a 90 % comparison coverage was constructed with CD-HIT (version 4.8.1). Gene abundances were estimated by quasi-mapping the quality-filtered reads from each sample to this gene catalog with Salmon, which outputs TPM and counts after resolving multi-mapped reads by an expectation–maximization procedure. Carbohydrate-active enzyme (CAZy) annotation was performed using the dbCAN2 metagenomic server (Hou et al., 2024).

### 2.4 Non-targeted metabolome analysis

Metabolite extraction from fermented grains was performed using a previously published protocol (Wang et al., 2022). Briefly, metabolites were extracted from 50 mg of freeze-dried fermented grains using 400 μL of an extraction solution (methanol:water = 4:1 *v*/v). The sample solution was ground for 5 min (−10 °C, 70 Hz) and then treated with ultrasound for 30 min (5 °C, 40 kHz). The sample was placed at −20 °C for 30 min, and the supernatant was removed for subsequent analysis. LC–MS analyses were performed on a UHPLC system coupled to a Q Exactive HF-X Orbitrap mass spectrometer (Thermo Fisher Scientific, Bremen, Germany) using an ACQUITY UPLC HSS T3 column (Waters Corporation, Milford, MA, USA). the elution gradient is provided in Table S1. Full MS and ddMS2 data were acquired in both positive and negative ESI modes. The mass spectrometry parameters are shown in Table S2. For each biological sample, one extract was prepared and analyzed with duplicate UHPLC–MS injections (technical replicates, *n* = 2 per sample). Pooled QC samples (equal aliquots of all extracts) were injected at regular intervals (every 10 injections), solvent blanks were included to monitor carryover, and injection order was randomized within and across groups. As described previously (Wang et al., 2022), LC–MS raw data were imported into Progenesis QI software (Waters Corporation, Milford, MA, USA) to obtain a data matrix containing the retention time, mass-to-charge ratio, and peak intensity. Subsequently, the data matrix was matched with the HMDB database (http://www.hmdb.ca/) to obtain metabolite information. Metabolites were differentially expressed between groups if they fulfilled the following standards: (1) *p* < 0.05 (*p* value obtained using a student's *t*-test) and (2) variable importance for the projection (VIP) > 1 based on orthogonal partial least squares discriminant analysis (OPLS-DA) (Qu et al., 2023). Such differentially expressed metabolites (DEMs) were deemed as potential biomarkers. The complete third-party analysis report, including parameter settings, QC diagnostics, and downloadable result tables/figures, is publicly available at: https://analysis.majorbio.com/metab/report_view/task_id/majorbio_323842 (task ID: majorbio_323842).

### 2.5 Statistical analysis and data visualization

The Mann–Whitney test was used to evaluate the differences in microorganisms and metabolites between the Dacha and Ercha fermented grains. Moreover, Spearman's rank correlation test was employed to determine the correlations among different microbial groups. Significant differences in β diversity were determined through permutational multivariate analysis of variance (PERMANOVA). *p* < 0.05 was considered statistically significant. Principal component analysis (PCA), principal coordinate analysis (PCoA), and graph plotting were performed using RStudio (version 4.3.2; Posit, PBC, Boston, MA, USA) and GraphPad Prism (version 10.2.3; GraphPad Software, San Diego, CA, USA).

## 3 Results

### 3.1 Microbial community diversity, composition, and differentially abundant taxa in dacha and Ercha fermented grains

**Fig. 2:**
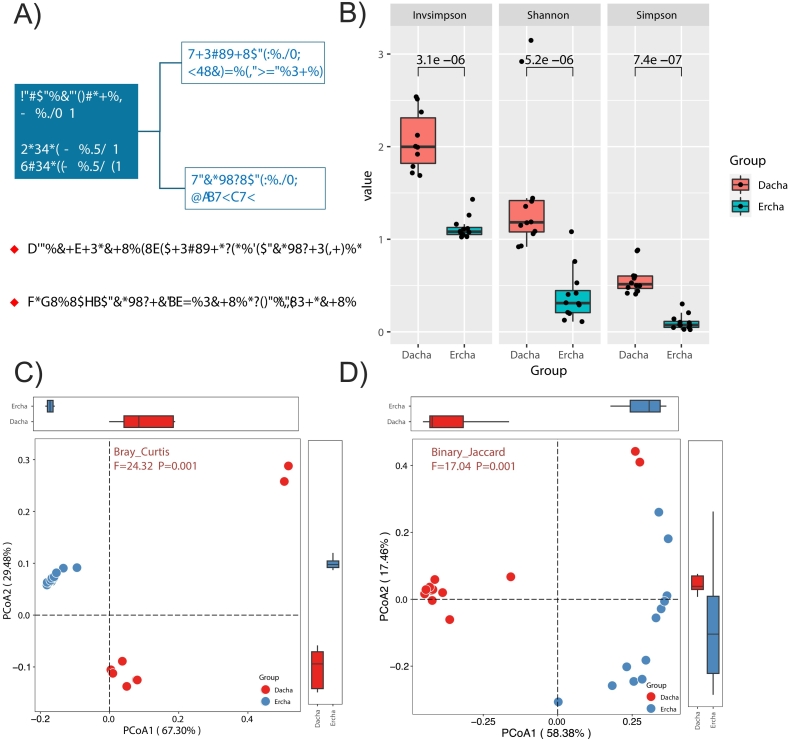
Microbial ⍺- and β- diversity of the Dacha and Ercha fermented grains. (A) Study design. Metagenomic sequencing and LC-MS/MS were used to characterize the microbiome and metabolome of 24 fermented grain samples (12 Dacha samples and 12 Ercha samples) from Xiangyang city, Hubei province (Discovery cohort). α-diversity was calculated based on the InvSimpson index, Shannon diversity index, and Simpson's diversity index (B). *p* values were calculated using the Wilcoxon rank sum test. β-diversity was estimated by PCoA based on Bray–Curtis (C) and Binary Jaccard distances (D) between the Dacha and Ercha groups. *p* values were calculated using two-sided unpaired student's *t*-tests. LC-MS/MS, liquid chromatography–tandem mass spectrometry; PCoA, principal coordinate analysis; PERMANOVA, permutational multivariate analysis of variance analysis.

This study examined 24 fermented grain samples of Fen-flavor Baijiu from Xiang Yang city, Hubei province. The detailed scheme of the study design is presented in Fig. 2A. Alpha-diversity indexes — including the InvSimpson index, Shannon diversity index, and Simpson's diversity index — were significantly lower in the Ercha fermented grains than in the Dacha fermented grains (*p* < 0.001) (Fig. 2B). Beta diversity in the Dacha and Ercha groups was calculated and visualized through PCoA using Bray–Curtis (Fig. 2C) and Binary Jaccard distances (Fig. 1D). The data demonstrated a significantly different distribution of microbiome structures between the Dacha and Ercha groups (both *p* < 0.001, PERMANOVA).

**Fig. 3:**
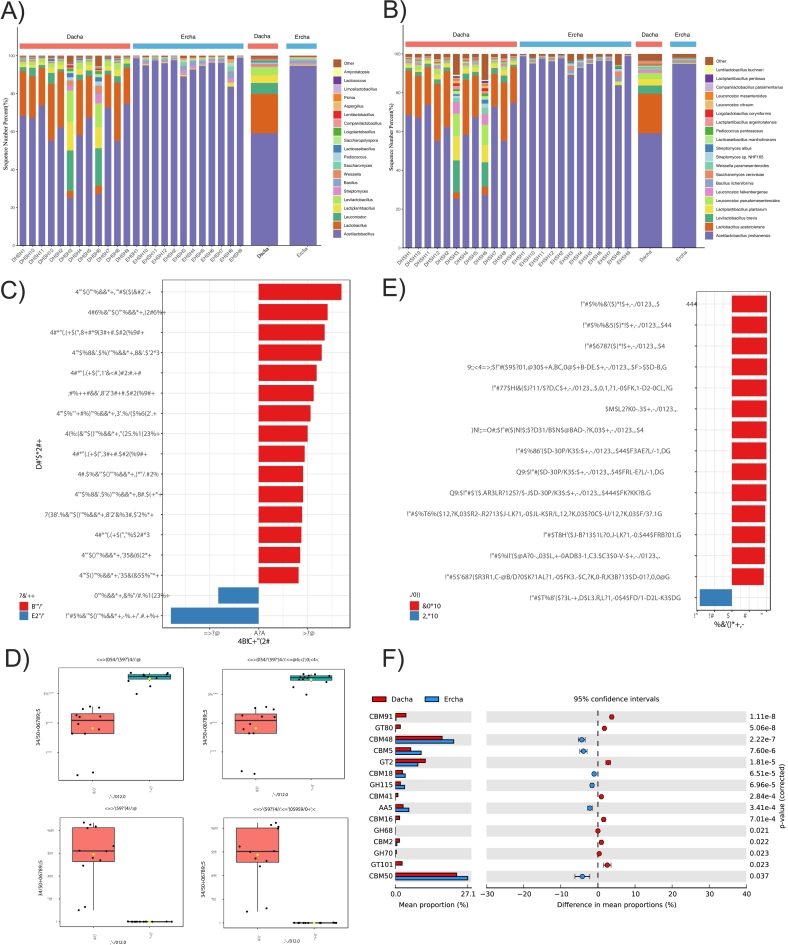
Taxonomic signatures and functional genes of the Dacha and Ercha microbiomes based on shotgun metagenomic sequencing. (A-B) Histogram showing the top 10 genera (A) and species (B) with the highest relative abundance. (C) The top 17 different species between Dacha and Ercha fermented grains were identified based on LefSe analysis following metagenomic sequencing (*n* = 12 for Dacha and n = 12 for Ercha). Logarithmic LDA score > 2.0, *p* < 0.05. (D) Box plot showing the taxonomic features of Dacha and Ercha fermented grains based on LDA score > 4.0 and *p* < 0.05. *p* values were calculated using the Wilcox test. (C) LefSe analysis showing the microbiota composition differences between the two groups at the species level (*p* < 0.05 and LDA > 2). E) LefSe analysis showing the difference in KEGG pathway abundance (*p* < 0.05 and LDA > 3). (F) STAMP analysis of functional differences in CAZy between the two groups. The results were filtered using an adjusted *p*-value of 0.05 [Bonferroni correction].

The microbiota of the fermented grains primarily consisted of bacteria (99.23 %), but small proportions of fungi (0.76 %) and viruses (0.01 %) were also detected. A total of 39 phyla, 1151 genera, and 3541 species were identified across the 24 samples. The major phyla were Bacillota (97.57 %), Actinobacteria (1.46 %), and Ascomycota (0.75 %) (Table S3). At the genus level, five genera exhibited mean relative abundance >1 %, led by *Acetilactobacillus* (76.76 %), followed by Lactobacillus (10.45 %), *Leuconostoc* (2.90 %), *Lactiplantibacillus* (2.22 %), and *Levilactobacillus* (2.18 %). Relative to Dacha, Ercha harbored a significantly higher abundance of *Acetilactobacillus* and significantly lower abundances of the other four genera (*p* < 0.05; Fig. 3A). At the species level, five taxa exceeded 1 % average relative abundance: *Acetilactobacillus jinshanensis* (76.81 %), *Lactobacillus acetotolerans* (10.22 %), *Levilactobacillus brevis* (2.17 %), *Lactiplantibacillus plantarum* (1.86 %), and *Leuconostoc pseudomesenteroides* (1.54 %) (Fig. 3B). The relative abundance of *A. jinshanensis* was strongly negatively correlated with those of *Levilactobacillus brevis*, *Lactiplantibacillus plantarum*, *Leuconostoc pseudomesenteroides*, and *Leuconostoc falkenbergense* (Supplementary Fig. 1). LEfSe identified 17 differentially abundant bacterial species between Dacha and Ercha (LDA > 2.0; *p* < 0.05; Fig. 3C). *Lactobacillus* and *Lactobacillus acetotolerans* showed the highest LDA scores in Dacha, whereas *Acetilactobacillus* and *A. jinshanensis* were most enriched in Ercha (all LDA > 4; *p* < 0.05; Fig. 3D).

### 3.2 KEGG pathway-based differences in microbial genes between dacha and Ercha fermented grains

The numbers of unigenes mapped to each KEGG/MetaCyc pathway are summarized in Table S4. Among metabolic pathways, the top three functions were PWY-7791 (cis-vaccenate biosynthesis), PWY-7663 (gondoate biosynthesis, anaerobic), and PWY-5384 (sucrose degradation IV via sucrose phosphorylase). We further profiled functional features across fermentation rounds using LEfSe (Fig. 3E). Dacha was enriched for pathways including UMP biosynthesis, O_antigen_building_blocks_biosynthesis, and fatty_acid_biosynthesis_initiation, whereas Ercha was characterized by enrichment in anerobic_respiration_I_(cytochrome_c).

We profiled carbohydrate-active enzyme families in Dacha and Ercha metagenomes. Multiple families differed between groups (FDR-adjusted *p* < 0.05; Fig. 3F; Table S5). The following families showed higher relative abundance in Dacha: CBM91, GT80, GT2, CBM41, CBM16, CBM51, GH68, CBM2, GH70, and GT101. In contrast, CBM48, CBM5, CBM18, GH115, AA5, and CBM50 were enriched in Ercha. Pairwise abundance correlations indicated that GH68 and GH70 were inversely related to GH115, CBM5, CBM50, CBM48, and AA5 (Supplementary Fig. 2).

### 3.3 Metabolomic alterations in dacha and Ercha fermented grains

**Fig. 4:**
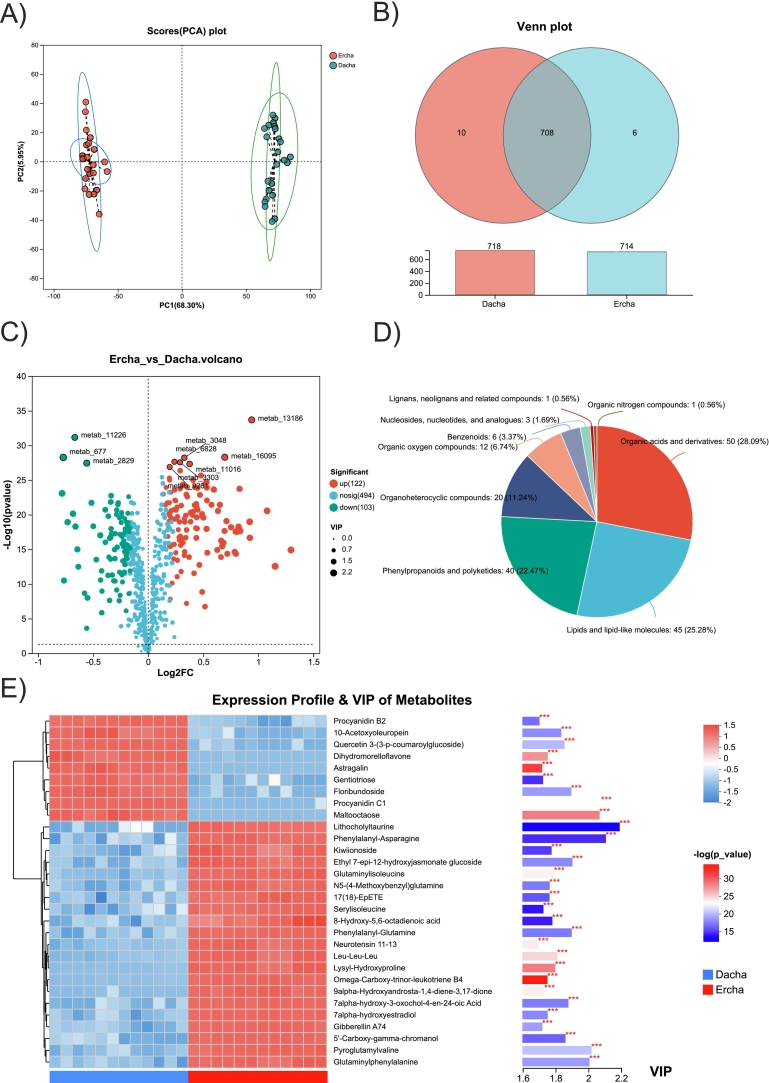
Differentially expressed metabolites between Dacha and Ercha fermented grains. (A) PCA plot showing the metabolomic distribution in the Dacha and Ercha samples in the anion plus cationic models. (B) Heat maps of the top 50 metabolites in the 24 samples. (C) Volcano plot showing upregulated and downregulated metabolites. (D) Histogram plot showing the number of upregulated and downregulated metabolites. (E) VIP scores of top 30 metabolites in Dacha and Ercha fermented grains. The selected metabolites showed VIP scores >1 on OPLS-DA. In the heat maps, red and blue boxes on the right indicate high and low abundance ratios, respectively, for the corresponding metabolites in Dacha and Ercha. (For interpretation of the references to colour in this figure legend, the reader is referred to the web version of this article.)

We conducted untargeted LC–MS metabolomics in positive and negative ion modes. Quality assessment showed tight clustering of pooled QC injections and stable instrument performance. Unsupervised PCA displayed clear separation between Dacha and Ercha in both modes (Fig. 4A). After preprocessing and annotation, 341 metabolites were identified in positive mode and 378 in negative mode. Presence–absence analysis indicated 10 metabolites detected only in Dacha (e.g., maltooctaose, dihydromorelloflavone, astragalin, procyanidin C1) and 8 detected only in Ercha (e.g., propylene glycol stearate, lysyl-hydroxyproline, lithocholyltaurine) (Fig. 4B).

In total, 225 differential metabolites were identified between groups (OPLS-DA VIP > 1 and FDR-adjusted *p* < 0.05), with 122 showing higher relative abundance in Ercha and 103 higher in Dacha (Fig. 4C). These metabolites mapped to nine HMDB superclasses, most frequently organic acids and derivatives (28.09 %), lipids and lipid-like molecules (25.28 %), phenylpropanoids and polyketides (22.47 %), and organoheterocyclic compounds (11.24 %) (Fig. 4D). The top 30 metabolites by VIP score and their group-specific abundance patterns are shown in Fig. 4E.

### 3.4 KEGG pathway overview of metabolite differences between dacha and Ercha fermented grains

**Fig. 5:**
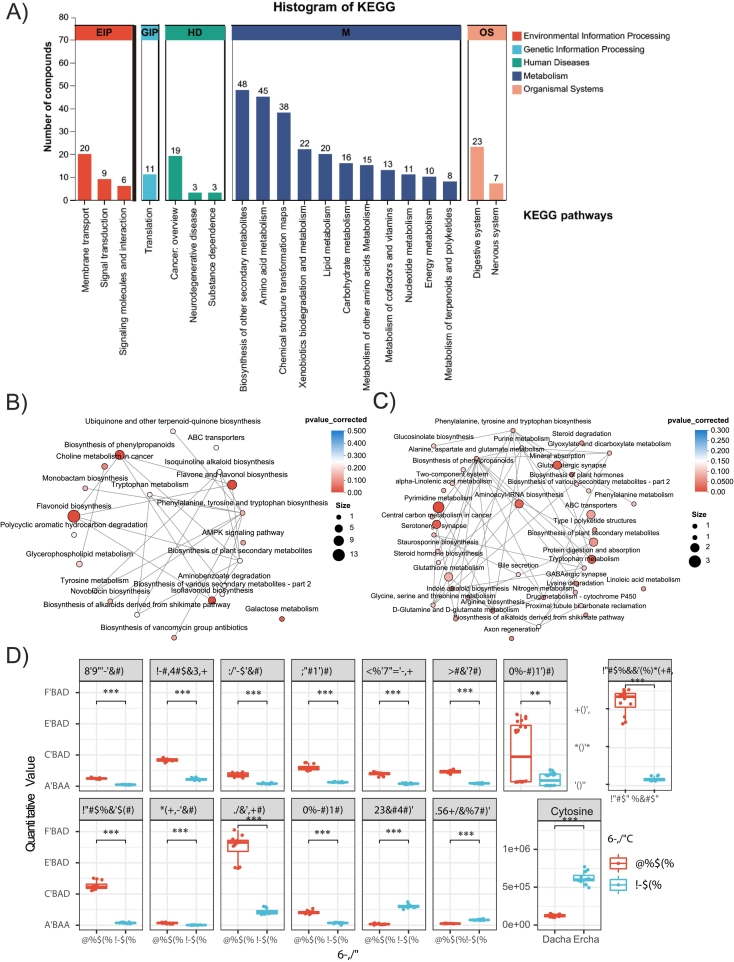
Metabolomics signatures of Dacha and Ercha fermented grains associated with flavonoid and pyrimidine biosynthesis, respectively. (A) Top 20 KEGG pathways associated with the identified metabolites. (B—C) KEGG pathway analyses of DEMs enriched in the Dacha group (B) and Ercha group (C). D) Box plot showing the abundance of the metabolites associated with flavonoid and pyrimidine biosynthesis in the Dacha and Ercha groups. *p* values were calculated using the Wilcox test.

Identified metabolites spanned 20 KEGG level-2 pathways. Within metabolism, the most represented terms included biosynthesis of other secondary metabolites, amino acid metabolism, chemical structure transformation maps, and xenobiotics biodegradation (Fig. 5A). Enrichment analysis of DEMs highlighted flavonoid biosynthesis and flavone/flavonol metabolism in Dacha, whereas pyrimidine metabolism was significantly enriched in Ercha (Fig. 5B–C). Because these KEGG pathway labels primarily refer to plant metabolic maps, we interpret the Dacha enrichment as higher levels of plant-derived flavonoids that were released and/or biotransformed during fermentation, rather than de novo microbial biosynthesis. In the flavonoid axis, 12 DEMs differed between groups, with epigallocatechin highest in Dacha (*p* < 0.01). For pyrimidine metabolism, cytidine, l-glutamine, and cytosine differed significantly, with cytosine highest in Ercha (*p* < 0.001) (Fig. 5D).

### 3.5 Associations between microbiota, CAZy functions, and flavonoid/pyrimidine metabolites between dacha and Ercha fermented grains

**Fig. 6:**
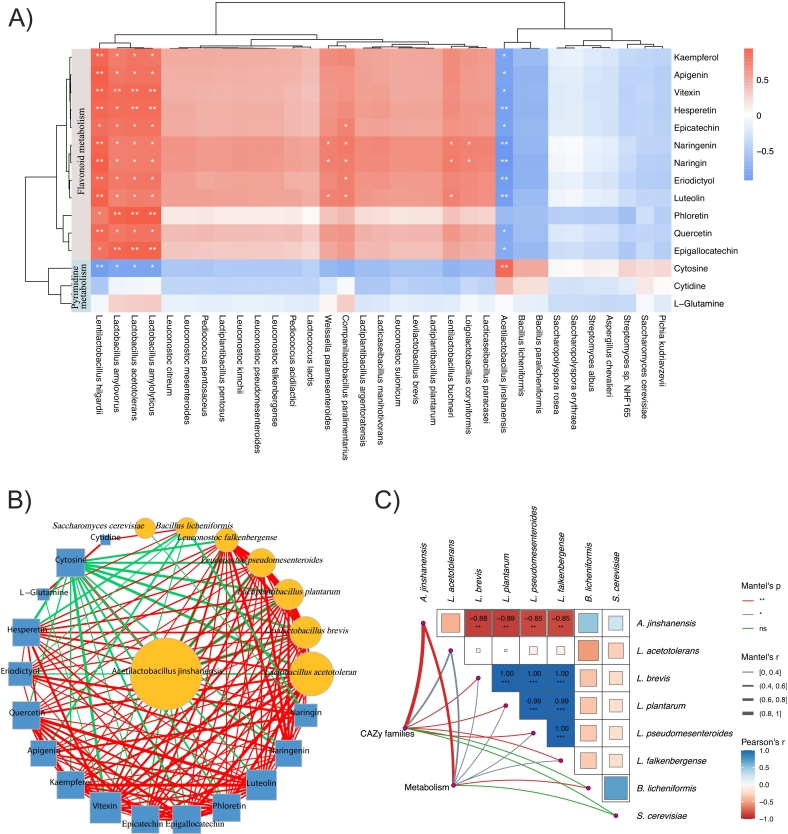
Integrated multi-omics analysis of Dacha and Ercha fermented grains. (A) Heatmap showing the associations between the microbiota and metabolites of the fermented grains. (B) The network of representatively significant and suggestive associations (*p < 0*.05, Pearson's analysis) among differentially abundant taxa and metabolites in the Dacha and Ercha groups. Nodes are colored according to the group (features increased or decreased versus the control). Lines connecting nodes indicate positive (red) or negative (gray) correlations. (C) Mantel tests and Pearson's correlation matrix showing representatively significant and suggestive associations among differentially abundant taxa, CAZys, and metabolites in the Dacha and Ercha groups. (For interpretation of the references to colour in this figure legend, the reader is referred to the web version of this article.)

To probe microbe–metabolite interactions underlying Dacha–Ercha differences, we correlated species with mean relative abundance >0.05 % against DEMs in flavonoid and pyrimidine pathways (Fig. 6A). Four taxa—*Lentilactobacillus hilgardii*, *Lactobacillus amylovorus*, *Lactobacillus acetotolerans*, and *Lactobacillus amylolyticus*—showed positive associations with multiple flavonoids (kaempferol, apigenin, vitexin, hesperetin, epicatechin, naringenin, naringin, eriodictyol, luteolin, phloretin, quercetin, epigallocatechin) and negative associations with cytosine, indicating a linkage between these LAB and flavonoid enrichment alongside lower pyrimidine levels. These associations are correlative and should not be interpreted as evidence of de novo bacterial production of flavonoids; they are more consistent with microbially mediated release and/or biotransformation of plant-derived compounds during fermentation. Consistent with community segregation, *Acetilactobacillus jinshanensis* (Ercha-enriched) was strongly negatively correlated with *Lactobacillus acetotolerans*, *Levilactobacillus brevis*, and *Lactiplantibacillus plantarum* (Dacha-enriched) (Fig. 6B). Mantel and Pearson analyses further supported significant and suggestive associations among differentially abundant taxa, CAZy families, and metabolites (Fig. 6C). Notably, flavonoid-related DEMs aligned more strongly with CAZy profiles than with individual species abundances overall, suggesting that carbohydrate-active functions may better capture metabolite differences between Dacha and Ercha than taxonomy alone.

### 3.6 Enzyme–taxon-based metabolic potential and pathway reconstruction in dacha and Ercha fermented grains

**Fig. 7:**
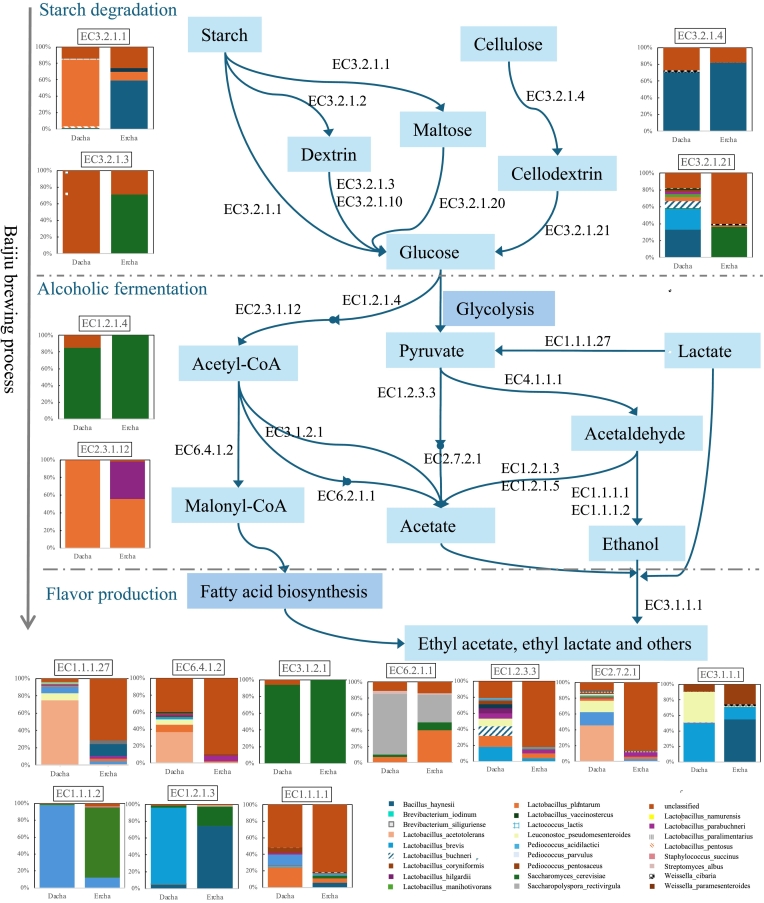
Schematic diagram showing the metabolism of ethanol, organic acids, and other flavor substances in the fermented grains during Fen-flavor Baijiu fermentation. Relevant enzymes and microorganisms involved in metabolism are listed. Only the top 10 microbes with the highest relative abundance are shown in the stacked bar chart. Other microorganisms have been grouped under “unclassified microorganisms.”

Guided by these associations, we mapped key pathways and enzyme–taxon linkages across major stages of Baijiu fermentation (starch/cellulose degradation, ethanol formation, and ester production) based on metagenome-detected genes (Fig. 7). Sorghum amylose/amylopectin are primarily hydrolyzed by α-amylase (EC 3.2.1.1) and β-amylase (EC 3.2.1.2). In our datasets, β-amylase genes were detected only in *Lactiplantibacillus plantarum*, while α-amylase genes were carried by *Bacillus haynesii*, *Lactiplantibacillus plantarum*, *Lactobacillus pentosus*, *Lactobacillus paralimentarius*, and *Streptomyces albus*. Among these carriers, *Lactiplantibacillus plantarum* predominated in Dacha, whereas *Bacillus haynesii* predominated in Ercha. Sorghum cellulose can be hydrolyzed by cellulolytic enzymes (e.g., endoglucanase, EC 3.2.1.4), which were mainly associated with *Bacillus haynesii*.

For alcohol fermentation, alcohol dehydrogenase (EC 1.1.1.1) and aldehyde reductase (EC 1.1.1.2) genes were prevalent, with dominant carriers including *Levilactobacillus brevis*, *Saccharomyces cerevisiae*, *Bacillus haynesii*, *Lactiplantibacillus plantarum*, and *Staphylococcus succinus*. Ethanol, acetic acid, and lactic acid can subsequently serve as precursors for ethyl acetate and ethyl lactate formation. Carboxylesterase (EC 3.1.1.1), a representative enzyme in esterification, was chiefly linked to *Leuconostoc pseudomesenteroides* and *Levilactobacillus brevis* in Dacha, and to *Bacillus haynesii* in Ercha. Overall, Dacha featured LAB-centered carbohydrate utilization and esterification potential, whereas Ercha showed stronger *Bacillus*-associated starch/cellulose hydrolysis alongside ethanol/ester formation.

## 4 Discussion

In contrast to that of other types of Baijiu, the fermentation process of Fen-flavor Baijiu is divided into two distinct rounds. The microbial community composition and variation during these two fermentation stages are crucial for the formation of flavor compounds and affect the overall quality of the liquor (Yu et al., 2023). In this study, we employed metagenomic and metabolomic analyses to comprehensively and scientifically evaluate the microbial community structure, functional characteristics, and metabolite composition of the fermented grains from both the stages of fermentation. Elucidating the metabolic functions of core microbes in these different fermented grains could be helpful for gaining deeper insights into the fermentation mechanisms of traditional fermented foods and enable potential improvements in future production processes.

Dacha fermented grains had a higher α-diversity than Ercha fermented grains (Fig. 2). This observation was consistent with previous findings regarding the microbial community structure of fermented grains from the Xinghuacun Fenjiu distillery and could be attributed to environmental factors, including nutrient limitations and reduced fermentation temperatures (Zhao et al., 2022). Bacteria were the predominant microbes in the fermented grains (99.23 %). *Lactobacillus acetotolerans* was the most abundant species in the Dacha group, and *A. jinshanensis* was the most abundant species in the Ercha group (Fig. 3). Previous studies have demonstrated that the bacterial abundance can increase from 58.2 % to 97.65 % by the end of fermentation, and only LAB typically survive until the end of the process (Chen et al., 2022; Huang et al., 2020). *Lactobacillus acetotolerans* was reported to be the absolute dominant strain after 22 days of fermentation, and its abundance increased rapidly in the later period of fermentation (Luo et al., 2023). LAB are resistant to low-oxygen, low-pH, and high-alcohol environments, and they can usually metabolize lactic acid, ethanol, and other compounds. The acidic environment created by the metabolic processes of these microbes can also inhibit the reproduction of pathogenic bacteria, spoilage bacteria, and toxigenic bacteria (Wu et al., 2021). *Acetilactobacillus* is a new genus of LAB that was discovered in 2020, with *A. jinshanensis* being the model species and the only strain identified within this genus (Yu et al., 2020). Recent studies have demonstrated that *A. jinshanensis* is widely distributed in fermentation systems such as vinegar, Luzhou-flavor Baijiu, and sauce-flavor Baijiu (Chen et al., 2024; Qian et al., 2021). Guan Tongwei et al. examined the microbial community structure of cellars used to ferment a Luzhou-flavor liquor from Sichuan for 6 and 30 years and found that the relative abundance of *A. jinshanensis* in the cellars fermented for 70 days after the 30-year period was as high as 38 % (Guan et al., 2020). This evidence suggests that *A. jinshanensis* may be better suited to the high-alcohol and low-oxygen conditions present in the Ercha fermented grains than *Lactobacillus acetotolerans*. Previously, a series of functional bacteria, including acid-resistant strains that produce high yields of bacteriocins and highly auto-soluble *Lactobacillus*, were isolated from fermented grains (Luan et al., 2023). These strains hold multifunctional purposes. Notably, some of the strains can be used to strengthen Daqu or directly applied during a certain stage of Baijiu fermentation to improve the fermentation process and the quality of Baijiu (Chen et al., 2023).

In this study, untargeted metabolomics showed that the metabolomic composition of Dacha differed from that of Ercha (Fig. 4A). Differences in metabolite profiles are often associated with variation in microbial community composition (e.g., taxonomic relative abundances and alpha/beta diversity) and functional potential (e.g., metagenomic pathway and CAZy gene profiles) across fermentation rounds (Huang et al., 2020). Through UHPLC-MS, we identified a total of 719 metabolites, among which 225 metabolites were identified as DEMs between the Dacha and Ercha groups. We also acquired ddMS2 in both ESI polarities and used spectral library matching (mzCloud, MassBank) to support metabolite annotations. As reported previously, unique compounds such as hexanal, 3-hydroxy-2-butanone, trans-2-pentenal, and ethyl hexanoate can be used to distinguish among different types of fermented grains at different stages of fermentation (Wang et al., 2024). Here, we noticed that 10 metabolites, including maltooctaose, dihydromorelloflavone, astragalin, and procyanidin C1, were specific to Dacha (Fig. 4b). The maltooctaose residue indicated that starch degradation remained insufficient during Dacha fermentation. Meanwhile, the detection of procyanidin C1 by chemosensors has previously been used for the comprehensive taste evaluation of wine (Yu et al., 2024). Where MS2 was unavailable or low quality, annotations were based on accurate mass and retention time matches in HMDB and are reported as putative (MSI Level 3) due to isomeric/isobaric ambiguity and limited RT transferability. Through the KEGG pathway enrichment analysis of metabolites, we found that 45 DEMs were associated with amino acid metabolism (Fig. 5A). Amino acids play a significant role in the deterministic assembly of fungal communities and affect the flavor compounds of Baijiu (Wei et al., 2023). Unlike the Ercha fermented grains, the Dacha fermented grains were enriched for 13 types of flavonoids and their precursors (Fig. 5B). Natural polyphenols such as flavonoids and organic acids have attracted immense attention owing to their anti-radiation, anti-oxidant, anti-aging, and anti-bacterial properties as well as their other biological activities. These compounds have also been found to reduce blood pressure and lipid levels (He et al., 2023; Sanjay et al., 2024). Additionally, research shows that the extrusion method is important for optimally extracting flavonoids from fermented grains and improving their antioxidant capacity(Orozco-Angelino et al., 2023).

Based on the observed relationships between microbial species and DEMs, as well as the CAZY annotations associated with the species and metabolites, we observed that LAB, particularly *Lactobacillus acetotolerans* and *A. jinshanensis*, were the most active microbes during Fen-flavor baijiu fermentation (Fig.6). In a previous study, HS-SPME-GC–MS analysis revealed that esters and alcohols, which were most abundant in the fermented grains at day 98, had a strong positive correlation with the succession of *Lactobacillus* communities (Tang et al., 2021). The fermentation of Fen-flavor Baijiu involves starch liquefaction, saccharification, alcohol fermentation, and flavor substance formation. Our results indicated that different LAB strains participated in different stages of fermentation. *Lactiplantibacillus plantarum* produced a large amount of α-amylase (EC 3.2.1.1) which is involved in starch degradation and was the main participant in the starch liquefaction of Dacha fermented grains. However, *Bacillus haynesii* largely replaced *Lactiplantibacillus plantarum* in the starch liquefaction process of Ercha fermented grains (Fig. 7), which may be related to the pH recovery in the Ercha fermented grains (Ağçeli, 2023; Xue et al., 2022). Meanwhile, we also found that *Bacillus haynesii* was a major producer of cellulolytic enzymes (EC 3.2.1.4). Recent studies have demonstrated the powerful cellulolytic capacity of *Bacillus haynesii*, which results from the enzymatic activity of all three cellulolytic enzymes (endoglucanase, extranooglucanase, and β-glucosidase) (Palit & Das, 2024). *Levilactobacillus brevis* and *Saccharomyces cerevisiae* can provide a variety of enzymes, including aldehyde dehydrogenase (EC.1.2.1.3), alcohol dehydrogenase (EC 1.1.1.1), and alcohol dehydrogenase (NADP(+)) (EC 1.1.1.2), which are involved in alcohol formation in fermented grains. The intensive inoculation of indigenous *Levilactobacillus brevis* and yeast into the fermentation system of Fen-flavor Baijiu can enhance the control of solid fermentation and accelerate the fermentation process (Chen et al., 2023). Additionally, LAB exhibit a positive correlation with the contents of acids, esters, alcohols, and other trace compounds in fermented grains and Baijiu, and play a key role in the formation of flavor substances (Xu et al., 2023). In this study, *Lactobacillus acetotolerans* and *Lactiplantibacillus plantarum* provided genes encoding enzymes such as L-lactate dehydrogenase (EC 1.1.1.27) and acetate kinase (EC 2.7.2.1) for acetic acid and ethyl acetate formation in fermented grains (Fig. 7). Fortified fermentation with *Lactobacillus acetotolerans* can significantly promote the formation of ethyl acetate and ethyl lactate in Baijiu (Li et al., 2024; Pang et al., 2021). These results enrich our understanding of the influence of LAB on Baijiu flavor compounds during fermentation and highlight ways to improve Baijiu flavor and fermentation processes through suitable LAB starter mixes that optimize fermentation outcomes.

## 5 Conclusion

This study performed, for the first time, a comprehensive analysis of both metagenomic and metabolomic data to investigate the evolution of core microorganisms and the mechanisms underlying flavor formation in fermented grains at different stages of Fen-flavor Baijiu fermentation. We found that LAB, particularly *Lactobacillus acetotolerans*, are the predominant bacteria during the late-stage fermentation of Fen-flavor baijiu. These microorganisms play a crucial role in promoting the saccharification of starch and the production of esters, acids, alcohols, and other flavor compounds. These findings offer valuable insights into the quality differences observed at various stages of Fen-flavor Baijiu production, which could enhance blending techniques and improve overall liquor quality. This research holds significant implications for the improvement of production quality and the industrial development of Fen-flavor Baijiu.

## CRediT authorship contribution statement

**Dingwu Qu:** Writing – review & editing, Writing – original draft, Software, Methodology, Investigation, Funding acquisition, Formal analysis, Data curation. **Yurong Wang:** Methodology, Investigation, Data curation. **Lubo Cao:** Methodology, Investigation. **Qiangchuan Hou:** Methodology, Investigation. **Zhongjun Liu:** Resources, Investigation. **Ji'an Zhong:** Resources, Investigation. **Zhuang Guo:** Writing – review & editing, Validation, Supervision, Project administration, Formal analysis, Conceptualization.

## Declaration of competing interest

The authors declare that they have no known competing financial interests or personal relationships that could have appeared to influence the work reported in this paper.

## Data Availability

Data will be made available on request.

## Appendix A Supplementary data

Supplementary material 1 Fig. S1. Correlation heatmap of dominant microbial species from fermented grians. Supplementary material 1

Supplementary material 2 Fig. S2. Correlation heatmap of Dacha and Ercha groups on carbohydrate-binding modules (CBMs), glycoside hydrolases (GHs), glycosyl transferases (GTs), and auxiliary activity (AA) enzyme. Supplementary material 2

Supplementary material 3 **Table S1.** Mobile Phase Elution Gradient. **Table S2.** Mass spectrometry parameters. **Table S3.** Microbial composition of Dacha and Ercha at the species level. **Table S4.** Metabolic pathway profile of microbial communities based on HUMAnN2 analysis. **Table S5.** Annotation of CAZy families with significant differences in relative abundance between Dacha and Ercha. Supplementary material 3
