# Supplementary figures and images for: Combined microbiome and metabolome analysis of Dacha and Ercha fermented grains of Fen-flavor Baijiu
Source: Food Chem (Oxf). 2025 Sep 11;11:100298. doi: 10.1016/j.fochms.2025.100298 (PMC12466285; doi:10.1016/j.fochms.2025.100298)

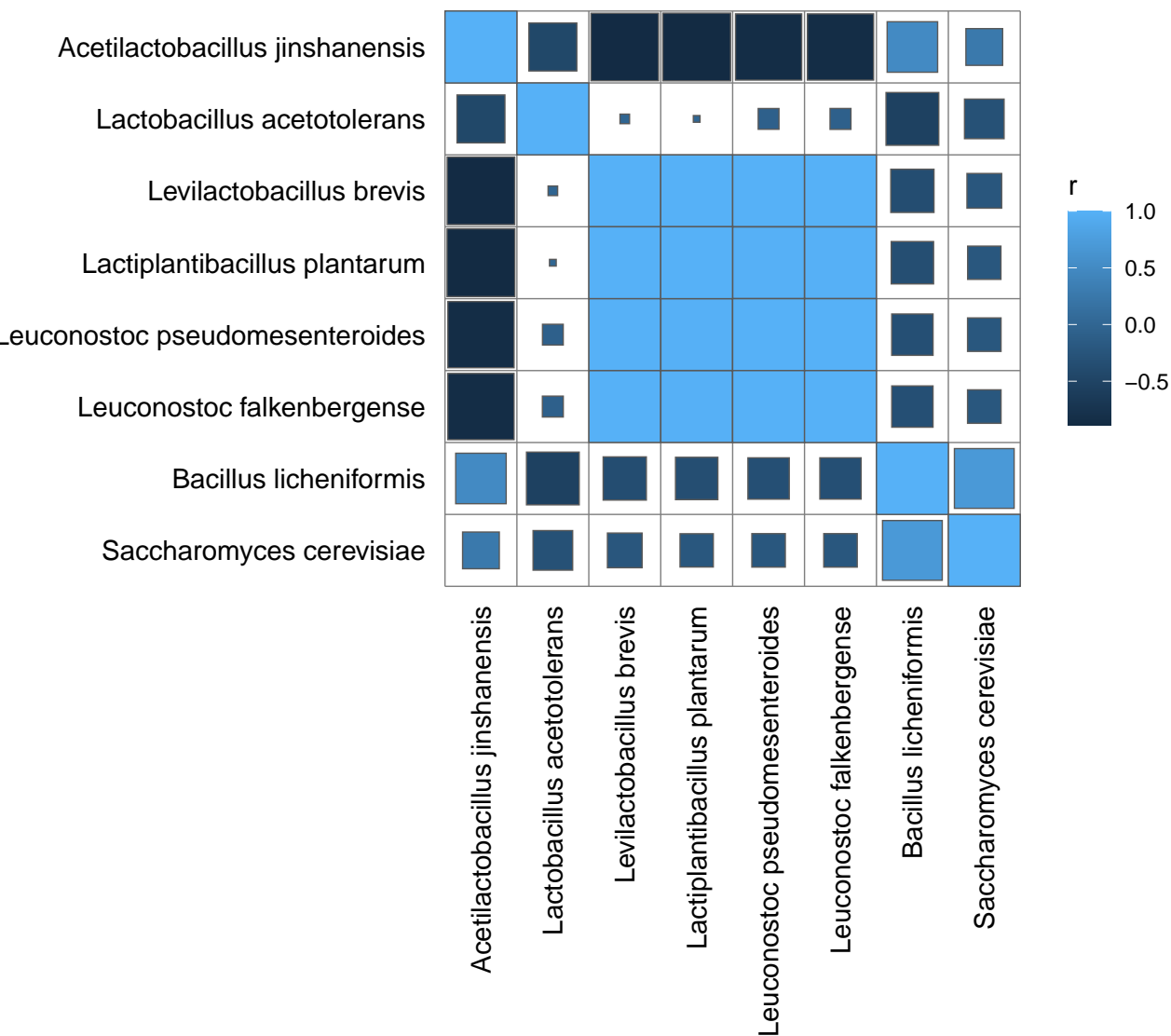

Supplement: Supplementary material 1 — Fig. S1. Correlation heatmap of dominant microbial species from fermented grians. [file mmc1.pdf]

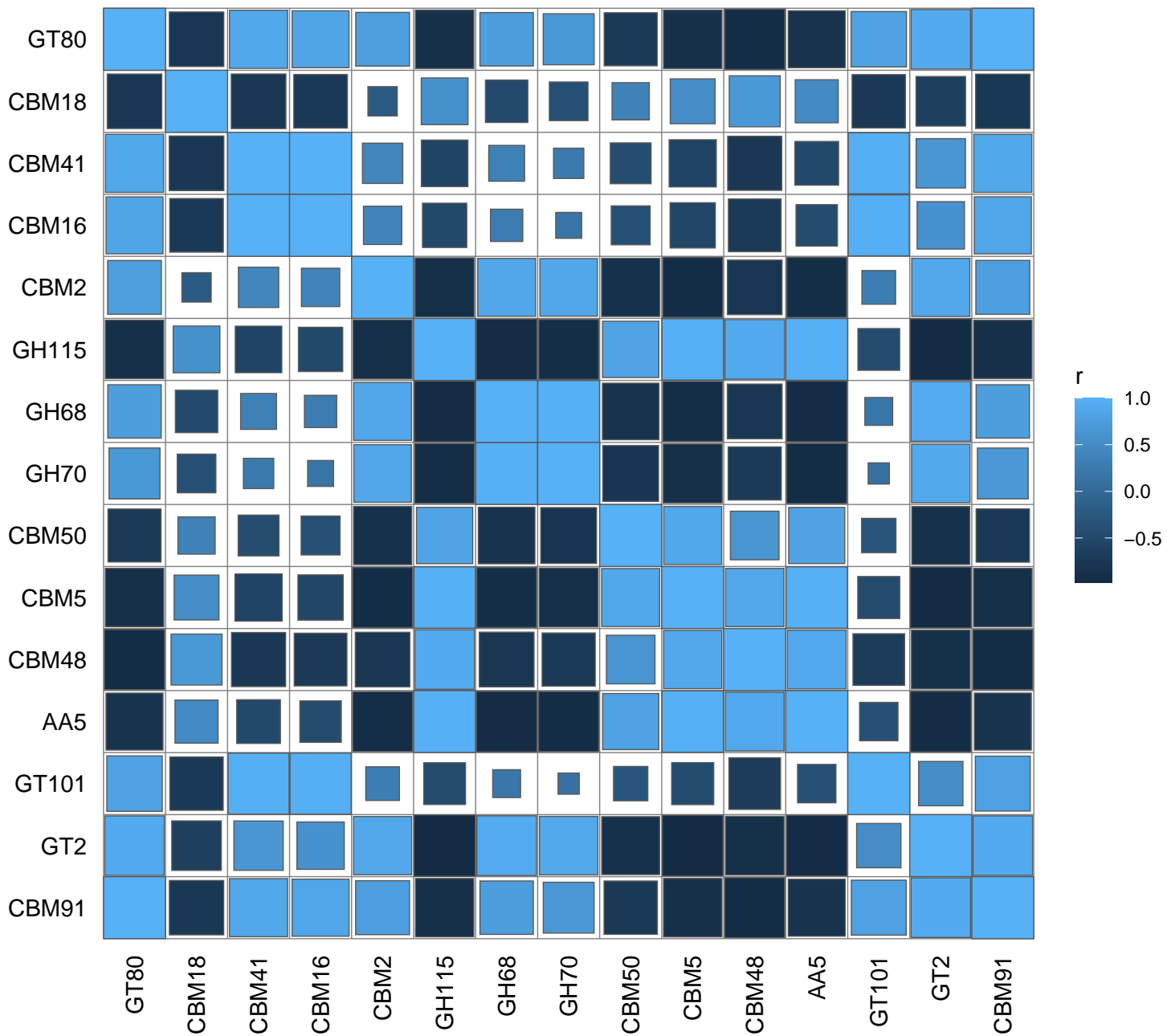

Supplement: Supplementary material 2 — Fig. S2. Correlation heatmap of Dacha and Ercha groups on carbohydrate-binding modules (CBMs), glycoside hydrolases (GHs), glycosyl transferases (GTs), and auxiliary activity (AA) enzyme. [file mmc2.pdf]
